# Recombinant Sapovirus Gastroenteritis, Japan

**DOI:** 10.3201/eid1305.070049

**Published:** 2007-05

**Authors:** Grant S. Hansman, Setsuko Ishida, Shima Yoshizumi, Masahiro Miyoshi, Tetsuya Ikeda, Tomoichiro Oka, Naokazu Takeda

**Affiliations:** * National Institute of Infectious Diseases, Tokyo, Japan; † Hokkaido Institute of Public Health, Hokkaido, Japan

**Keywords:** Sapovirus, genogroup, genotype, recombinant, polymerase gene, capsid protein, Japan, letter

**To the Editor:** Sapovirus and norovirus are causative agents of gastroenteritis in children and adults. Norovirus is the most important cause of outbreaks of gastroenteritis, whereas only a few outbreaks of sapovirus have been reported (*1*,*2*). On the basis of complete capsid gene sequences, sapovirus can be divided into 5 genogroups, among which GI, GII, GIV, and GV infect humans, whereas sapovirus GIII infects porcine species.

We report 2 outbreaks of gastroenteritis in Hokkaido, Japan. The first outbreak (A) occurred at a college from May 29 to June 2, 2000. A total of 12 persons (11 students and 1 teacher) reported symptoms of gastroenteritis (nausea, vomiting, stomachache, diarrhea, and fever); 11 stool specimens were collected from days 1 to 7 after onset of illness (Table). These specimens were negative for norovirus (data not shown), but 5 were positive for sapoviruslike viruses by electron microscopy (Table).

**Table:** Analysis of 18 stool specimens for sapovirus during 2 outbreaks of gastroenteritis, Japan*

Outbreak, specimen	Date of illness onset	EM	Nested RT-PCR	Real-time PCR (cDNA copies/g stool)
A				
Yak1	Jun 2, 2000	–	+	7.47 × 10^9^
Yak2	May 29, 2000	–	–	–
Yak3	May 30, 2000	–	–	–
Yak4	May 31, 2000	+	+	6.55 × 10^9^
Yak5	Jun 1, 2000	–	+	9.38 × 10^8^
Yak6	Jun 1, 2000	–	+	1.30 × 10^8^
Yak7	May 29, 2000	–	+	1.46 × 10^9^
Yak8	May 29, 2000	+	+	2.78 × 10^10^
Yak9	Jun 1, 2000	+	+	3.00 × 10^9^
Yak10	Jun 1, 2000	+	+	2.05 × 10^9^
Yak11	Jun 1, 2000	+	+	5.36 × 10^5^
B				
Nay1	Feb 17, 2005	NT	+	1.65 × 10^10^
Nay2	Feb 14, 2005	NT	+	1.82 × 10^9^
Nay3	Feb 18, 2005	NT	+	1.14 × 10^9^
Nay4	Feb 17, 2005	NT	+	5.41 × 10^10^
Nay5	Feb 16, 2005	NT	+	5.26 × 10^10^
Nay6	Feb 18, 2005	NT	+	2.50 × 10^10^
Nay7	Feb 17, 2005	NT	+	2.38 × 10^10^

*EM, electron microscopy; RT-PCR, reverse transcription–PCR; NT, not tested.

The 11 specimens were then examined for sapovirus by using nested reverse transcription–PCR (RT-PCR) as described (*3*). A total of 9 (82%) of 11 specimens were positive for sapovirus. Sequence analysis showed that these 9 viruses had 100% nucleotide identity and likely represented the same sapovirus strain (termed Yak2 strain, GenBank accession no. AB046353). To determine the number of cDNA copies per gram of stool, we performed real-time RT-PCR as described (*4*). The number of sapovirus cDNA copies ranged from 5.36 × 10^5^ to 7.47 × 10^9^/g stool (median 5.49 × 10^9^ copies/g stool) (Table).

The second outbreak (B) occurred at a kindergarten from February 1 to 22, 2005. A total of 23 persons (15 children and 8 adults) reported symptoms of gastroenteritis (nausea, vomiting, stomachache, diarrhea, and fever); 7 stool specimens were collected (Table). These specimens were negative for norovirus (data not shown), but all were positive for sapovirus by nested RT-PCR. The 7 sequences from this outbreak had 100% nucleotide identity and likely represented the same sapovirus strain (termed Nay1 strain, GenBank accession no. EF213768). The number of sapovirus cDNA copies ranged from 1.14 × 10^9^ to 5.41 × 10^10^/g stool (median 2.50 × 10^10^ copies/g stool) (Table).

One positive sapovirus specimen from each outbreak was subjected to further sequence analysis in which a single overlapping PCR fragment covering the partial polymerase gene and capsid gene was amplified. The Yak2 and Nay1 sequences shared ≈71% nucleotide identity for this fragment and likely represented different sapovirus strains. The Yak2 sequence closely matched sapovirus GIV Ehime1107 and SW278 sequences (GenBank accession nos. DQ058829 and AY237420, respectively) and had 98% and 97% nucleotide identity for the entire fragment, respectively (*5*). The Nay1 sequence closely matched the sapovirus GII C12 sequence (AY603425) and had 91% nucleotide identity for the entire fragment.

The Nay1 sequence closely matched the C12 sequence, which was detected in Osaka, Japan, in 2001 (*6*), whereas the Yak2 sequence closely matched the Ehime1107 sequence, which was detected in Matsuyama, Japan, in 2002 (*5*) and the SW278 sequence, which was detected in Sweden in 2003 (*1*). We recently described the C12 strain as intragenogroup recombinant sapovirus strain (*6*), whereas the Ehime1107 and SW278 strains were described as intergenogroup recombinant sapovirus strains (*5*). Our results indicate that recombination sites in intragenogroup and intergenogroup recombinant sapovirus strains were at the polymerase and capsid junction (*5**,**6*). Sapovirus Sydney53 (DQ104360) and Sydney3 strains (DQ104357), which were detected in Australia from August 2001 to August 2004 (*7*), closely matched C12 and Ehime1107/SW278 sequences, respectively. These results showed that recombinant sapovirus strains are stable in the environment and may be globally distributed. Our findings also suggest a changing distribution of sapovirus-associated gastroenteritis in Hokkaido because different sapovirus GI strains were predominant sapovirus strains that caused causing outbreaks of gastroenteritis in Hokkaido (*8*,*9*).

In a recent study, the number of norovirus cDNA copies per gram of stool specimen was analyzed and a discrepancy was found between the different norovirus genogroups (*10*). Chan et al. found that noroviruses GI and GII showed medians of 8.4 × 10^5^ and 3.0 × 10^8^ copies/g of stool specimen, respectively, and speculated that increased viral loads were caused by higher transmissibility of norovirus GII strains (*10*). Our results showed that sapovirus GII Nay1 and GIV Yak2 strains showed higher viral loads than norovirus GII strains. These results suggest that a high degree of shedding of sapovirus GII Nay1 and GIV Yak2 strains may have caused the outbreak of gastroenteritis. However, to elucidate this suggestion, further studies are needed with other sapovirus strains.
